# Activation of the mitochondrial unfolded protein response promotes longevity and dopamine neuron survival in Parkinson’s disease models

**DOI:** 10.1038/s41598-017-16637-2

**Published:** 2017-11-27

**Authors:** Jason F. Cooper, Emily Machiela, Dylan J. Dues, Katie K. Spielbauer, Megan M. Senchuk, Jeremy M. Van Raamsdonk

**Affiliations:** 1 Laboratory of Aging and Neurodegenerative Disease, Center for Neurodegenerative Science, Van Andel Research Institute, Grand Rapids, MI USA; 2 Department of Neurology and Neurosurgery, McGill University, Montreal, Quebec, Canada; 3 Metabolic Disorders and Complications Program, and Brain Repair and Integrative Neuroscience Program, Research Institute of the McGill University Health Centre, Montreal, Quebec, Canada

## Abstract

While the pathogenesis of Parkinson’s disease (PD) is incompletely understood, mitochondrial dysfunction is thought to play a crucial role in disease pathogenesis. Here, we examined the relationship between mitochondrial function and dopamine neuron dysfunction and death using *C. elegans* mutants for three mitochondria-related genes implicated in monogenic PD (*pdr-1/PRKN, pink-1/PINK1* and *djr-1.1/DJ-1*). We found that *pdr-1* and *pink-1* mutants exhibit deficits in dopamine-dependent behaviors, but no loss of dopamine neurons, while *djr-1.1* mutants showed an increased sensitivity to oxidative stress. In examining mitochondrial morphology and function, we found that *djr-1.1* mutants exhibit increased mitochondrial fragmentation leading to decreased rate of oxidative phosphorylation and ATP levels. *pdr-1* and *pink-1* mutants show an accumulation of dysfunctional mitochondria with age, which leads to activation of the mitochondrial unfolded protein response (mitoUPR). Preventing the upregulation of the mitoUPR with a deletion in *atfs-1* results in decreased lifespan and dopamine neuronal loss in *pdr-1* and *pink-1* mutants but not in wild-type worms. Overall, our results suggest that mutations in *pdr-1* and *pink-1* cause the accumulation of dysfunctional mitochondria, which activates the mitoUPR to mitigate the detrimental effect of these mutations on dopamine neuron survival.

## Introduction

Parkinson’s disease (PD) is the second most common neurodegenerative disease currently affecting as many as 10 million individuals worldwide. Clinically, PD is characterized by deficiencies in movement including bradykinesia, postural instability, resting tremor, rigidity, and a shuffling gait that become progressively worse through the course of disease. Patients also typically experience non-motor symptoms such as dementia, sleep disturbances, and depression. In the brain, PD patients exhibit the loss of dopamine neurons in the substantia nigra (SN), which is part of the basal ganglia. At the cellular level, neurons show accumulation of α-synuclein-containing protein aggregates known as Lewy Bodies.

While the pathogenesis of PD is incompletely understood, accumulating evidence suggests that mitochondrial dysfunction is a crucial step in disease pathogenesis. First, PD patients exhibit deficits in mitochondrial electron transport chain function^1^. Second, it has been shown that toxins that affect mitochondrial function, such as MPTP or rotenone, can selectively kill dopamine neurons^2,3^. As a result, these mitochondrial toxins have been used to model PD^4^. Third, genes involved in mitochondrial function have been shown to cause monogenic forms of PD, including *PRKN*, *PINK1* and *DJ-1* ^5–9^.

Parkin, encoded by *PRKN* in humans and *pdr-1* in *C. elegans*, is a component of the E3 ubiquitin ligase multiprotein complex that tags proteins for degradation in the ubiquitin-proteasome system. Under normal conditions, Parkin resides in the cytoplasm and ubiquitinates a wide variety of cytosolic targets^10,11^. However, under conditions of mitochondrial stress, Parkin can move to the mitochondria to target mitochondrial outer membrane proteins^12^. Notably, Parkin has been shown to work with PINK1 (PTEN-induced putative kinase 1) to control mitophagy, the process by which damaged mitochondria are degraded^13,14^.

PINK1, which is encoded by *pink-1* in *C. elegans*, is a kinase that is localized to the mitochondria. While PINK1 is degraded under normal conditions, when the mitochondrial membrane becomes depolarized due to stress, the degradation of PINK1 is prevented thereby allowing PINK1 to recruit the cellular machinery necessary to recycle defective mitochondria^15,16^. PINK1, the first known ubiquitin-kinase, recruits autophagy receptors, expediting the ability of Parkin to tag defective mitochondria with ubiquitin to initiate mitophagy^17^.

DJ-1 is a highly conserved protein^5^ that has been proposed to play a role in oxidative stress as a redox-sensitive chaperone^18–20^. The protein product of DJ-1 is a deglycase that has been found to localize to the mitochondria^21^. In *C. elegans*, DJ-1 is encoded by the *djr-1.1* and *djr-1.2* homologs, which have been shown to be primarily expressed in the intestine and in neurons, respectively^22^. Interestingly, DJ-1 has been shown to interact with parkin and PINK1 to promote protein degradation^23^.

In this work, we provide a thorough characterization of *C. elegans* models with deletions in mitochondrial-related genes that have been implicated in PD, and explore the relationship between mitochondrial function and phenotypic deficits. We find that *pdr-1, pink-1* and *djr-1.1* deletion mutants all exhibit mild phenotypes despite clear deficits in mitochondrial function. Our results suggest that there is an accumulation of dysfunctional mitochondria in *pdr-1* and *pink-1* mutants that leads to the activation of the mitochondrial unfolded protein response (mitoUPR). This response mitigates the detrimental effects of the PD mutations and protects against the loss of dopamine neurons.

## Results

### *pdr-1* and *pink-1* mutants display deficits in dopamine-dependent behaviors in absence of neurodegeneration

**Figure 1:**
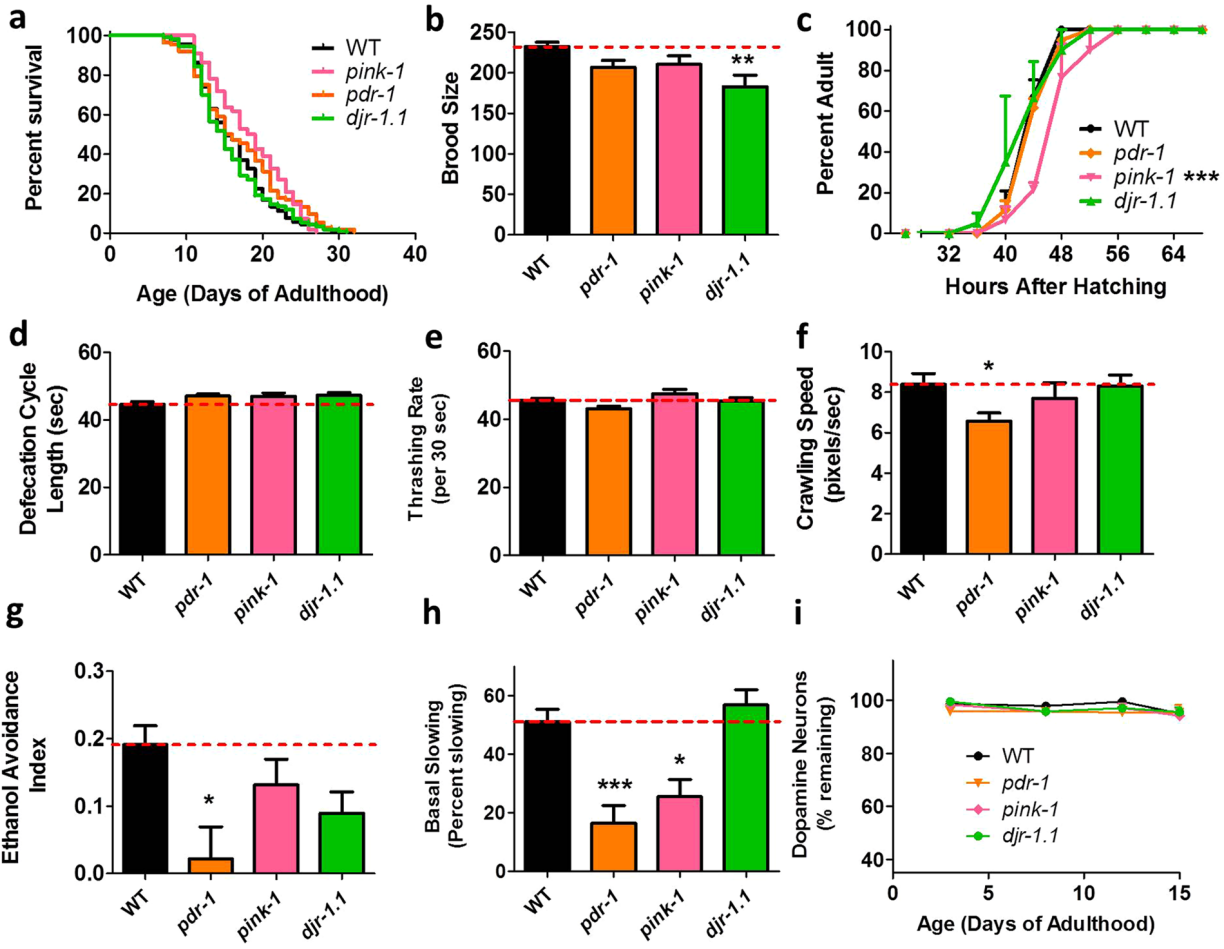
*p* *dr-1* and *pink-1* mutants exhibit deficits in dopamine-dependent behaviors. (**a**) Lifespan is not decreased in *pdr-1, pink-1* or *djr-1.1* mutants. (**b**) Fertility, as measured by self-brood size, is decreased in *djr-1.1* worms. (**c)** *pink-1* mutants develop significantly slower than wild-type worms. (**d**) Defecation cycle length is not affected in any of the mutants. (**e**) Movement in liquid (thrashing) is also unaffected. (**f**) *pdr-1* mutants show a mild deficit in crawling speed on solid plates. (**g**) All three mutants exhibit a trend towards decreased ethanol avoidance, which only reaches significance in *pdr-1* mutants. (**h**) Both *pdr-1* and *pink-1* mutants have a deficit in basal slowing. (**i**) Despite showing deficits in dopamine-dependent behaviors there is no evidence for neuronal loss in *pdr-1, pink-1* or *djr-1.1* mutants. This indicates that the deficits in dopamine-dependent behavior results from neuronal dysfunction, not neuronal loss. Error bars indicate SEM. *p < 0.05, ***p < 0.001.

To determine whether mutations in mitochondria-related genes that have been implicated in PD cause quantifiable deficits that could be used as outcome measures to identify disease modifiers, we characterized measures of general health and dopamine-dependent behavior in *pdr-1, pink-1* and *djr-1.1* worms. All three strains are deletion mutants thereby modeling the loss of function mutations present in the human disease. We found that lifespan was not decreased in any of the PD mutants (Fig. 1a). Decreased fertility, slow post-embryonic development time or a slow defecation cycle length can be indications that a worm is unhealthy. We found that fertility, as measured by brood size, is decreased in *djr-1.1* mutants (Fig. 1b), while *pink-1* mutants exhibit slow post-embryonic development (Fig. 1c). All three mutants exhibited a normal rate of defecation (Fig. 1d).

Next, we sought to determine if the PD mutations would affect movement, as this is a key feature of the human disease. Movement was assessed both in liquid, by determining the thrashing rate, and on solid plates by measuring crawling speed. In both cases, movement was assessed in an unbiased manner using video-tracking and computer analysis. We found that all of the PD mutants have a normal thrashing rate (Fig. 1e) and that *pdr-1* mutants have a mild movement deficit in crawling (Fig. 1f).

Finally, we tested behaviors that have previously been shown to be dependent on intact dopaminergic signaling, including ethanol avoidance and basal slowing. First, we verified that these behaviors are dopamine-dependent and absent in dopamine-depleted *cat-2* tyrosine hydroxylase mutants (Supplementary Fig. S1). In testing the PD mutants, we found that all three strains showed a trend towards decreased ethanol avoidance behavior, but the difference only reached significance in *pdr-1* worms (Fig. 1g). In the basal slowing assay, both *pdr-1* and *pink-1* worms showed a significant deficit in slowing (Fig. 1h).

In order to determine whether the change in dopamine-dependent behavior is caused by a loss of dopamine neurons, we crossed *pdr-1, pink-1* and *djr-1.1* worms to worms expressing GFP under the dopamine-specific *dat-1* promoter such that we could visualize neurons *in vivo* throughout the animal’s lifespan. We found that even at day fifteen of adulthood these mutant strains exhibit no loss of dopamine neurons (Fig. 1i). This suggests that the deficit in dopamine-dependent behaviors in *pdr-1* and *pink-1* mutants is caused by neuronal dysfunction, not neuronal death.

As a significant deficit in dopamine-dependent behavior was not observed in *djr-1.1* worms, we hypothesized that this may be due to the presence of *djr-1.2*, which is primarily expressed in neurons^22^. Accordingly, we characterized *djr-1.2* and *djr-1.1;djr-1.2* worms. As with *djr-1.1* worms, we did not observe a significant deficit in basal slowing or any evidence of dopamine neuron loss in either strain (Supplementary Fig. S2).

### *pdr-1* and *pink-1* mutants accumulate dysfunctional mitochondria with age

**Figure 2:**
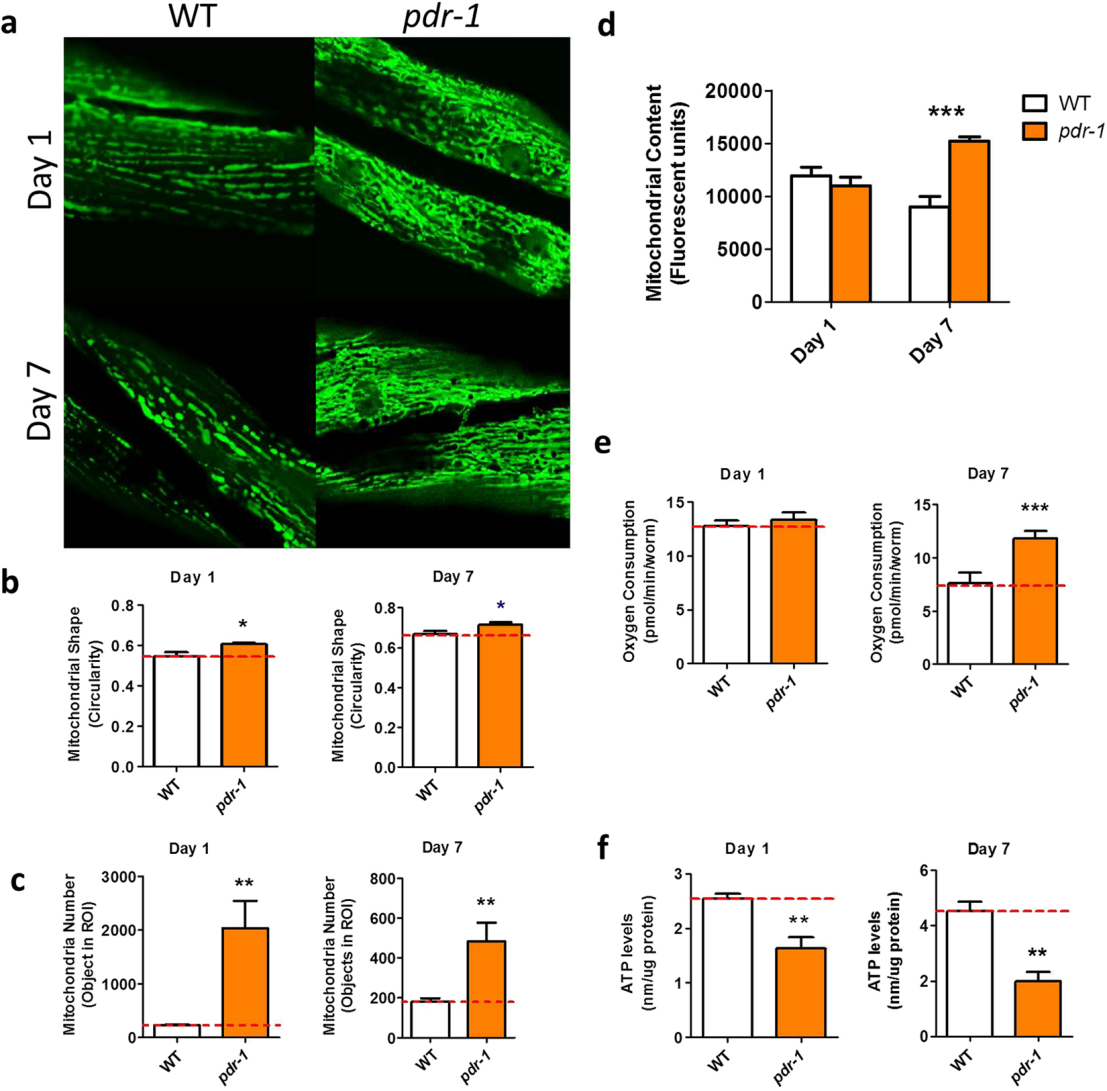
*pdr-1* mutants show an accumulation of dysfunctional mitochondria with age. (**a**) Mitochondria in *pdr-1* mutants exhibit abnormal morphology. (**b**) Quantification of mitochondrial shape reveals a significant increase in circularity in *pdr-1* mitochondria compared to wild-type. (**c)** *pdr-1* mutants also show a marked increase in mitochondrial number. (**d**) *pdr-1* mutants show an accumulation of mitochondria with age as indicated by increased mitochondrial content. The increase in mitochondrial content results in an increase in oxygen consumption (**e**), but a decrease in ATP levels (**f**). Error bars indicate SEM. *p < 0.05, **p < 0.01, ***p < 0.001.

Having observed deficits in dopamine-dependent behavior, we next sought to determine whether these deficits were associated with alterations in mitochondrial form and function. To visualize mitochondrial morphology we utilized a fluorescent reporter strain in which GFP is targeted to the mitochondrial matrix of body wall muscle cells (*P*_*myo-3*_ *::mitoGFP*)^24^. Images of mitochondria were collected at day 1 and day 7 of adulthood and quantified. In wild-type worms, mitochondria in the body wall muscle exhibit a very regular pattern consisting of many parallel tracts of elongated mitochondria (Fig. 2a). This pattern is maintained at day 7 of adulthood, although total mitochondrial content is decreased. In contrast, *pdr-1* worms exhibit less tubular mitochondria (increased circularity Fig. 2b) and have an increased abundance of mitochondria compared to wild-type worms (Fig. 2c,d), indicating that *pdr-1* worms accumulate mitochondria with age. As a control for increased mitochondrial fragmentation, we examined mitochondrial morphology in *fzo-1* worms, which have a deletion mutation that disrupts the outer mitochondrial membrane fusion protein FZO-1. As expected, these worms exhibit fragmented mitochondria at day 1 of adulthood and these mitochondria have increased circularity (Supplementary Fig. S3).

We then asked how the observed differences in mitochondrial morphology affect mitochondrial function by measuring the rate of oxidative phosphorylation and ATP levels. It has previously been shown that a more highly fused mitochondrial network can be more efficient at generating ATP^25,26^. The rate of oxidative phosphorylation was determined by measuring oxygen consumption using a Seahorse extracellular flux analyzer, while ATP levels were measured utilizing a luciferin-based assay. We found that *pdr-1* mutants exhibit increased oxygen consumption (Fig. 2e) and decreased ATP levels (Fig. 2f) compared to wild-type worms. The increase in oxygen consumption is consistent with the mitochondrial accumulation observed in *pdr-1* worms. The fact that ATP levels are decreased, despite an increased rate of oxidative phosphorylation, suggests that *pdr-1* mutants are accumulating dysfunctional mitochondria, and is consistent with the known role of Parkin in mitophagy.

**Figure 3:**
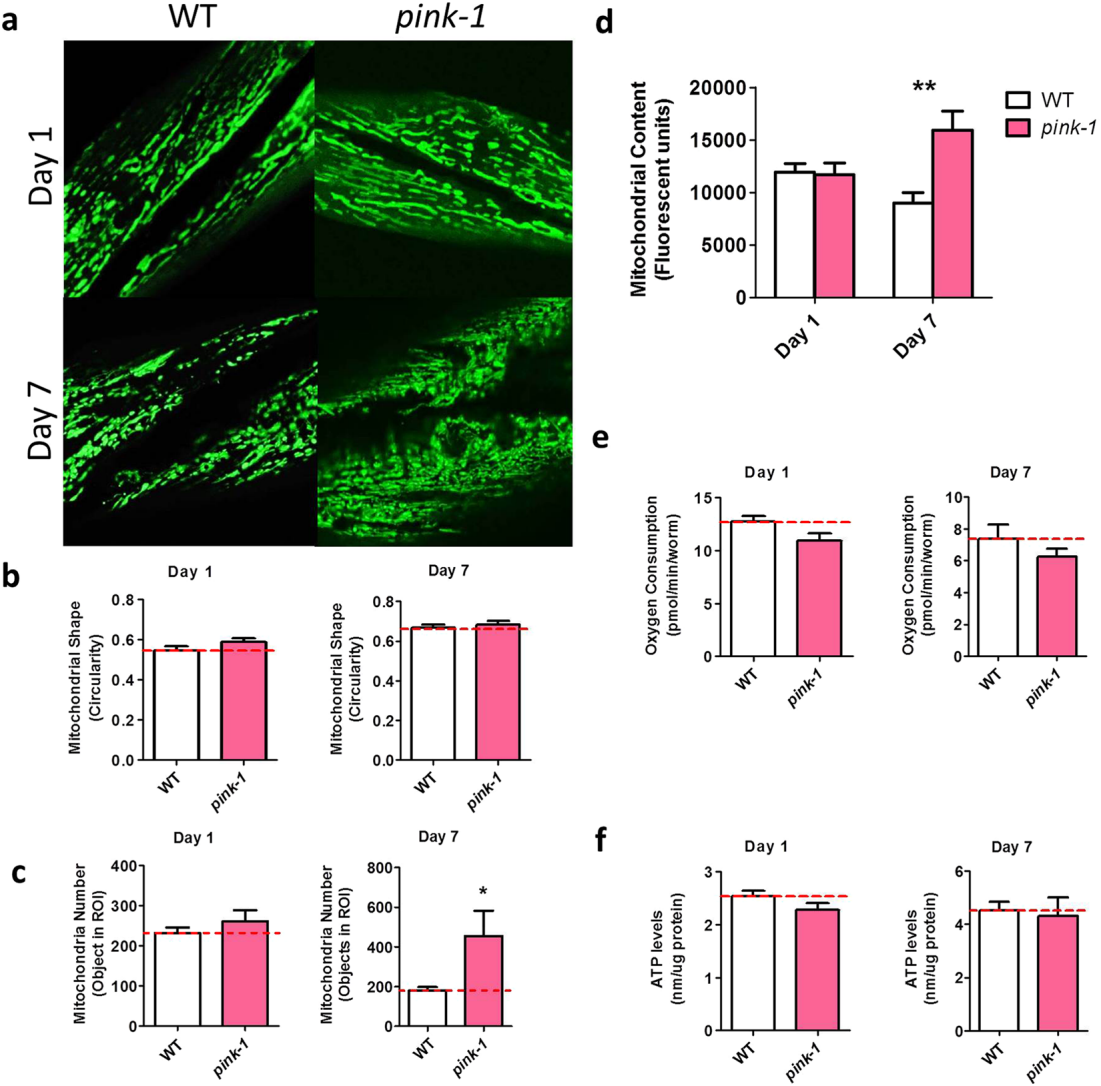
*pink-1* mutants show an accumulation of mitochondria with age. (**a**) Mitochondria in *pink-1* mutants exhibit abnormal morphology especially at the aged time point. While there was no difference in mitochondrial shape (**b**), *pink-1* show an increase in mitochondrial abundance with age (**c**,**d**). Nonetheless, oxygen consumption (**e**) and ATP levels (**f**) do not differ from wild-type worms. This suggests that the mitochondria in *pink-1* worms have a reduced rate of oxidative phosphorylation compared to wild-type worms but that the ATP produced per amount of oxygen consumed is not decreased. Error bars indicate SEM. *p < 0.05, **p < 0.01, ***p < 0.001.

As with *pdr-1* mutants, we found that mitochondria in *pink-1* worms exhibit an accumulation of mitochondria with increasing age (Fig. 3a,c,d). However, in contrast to *pdr-1* worms, the shape of *pink-1* mitochondria was not different from wild-type (Fig. 3b). Measurement of oxygen consumption revealed that although *pink-1* worms show a similar increase in mitochondrial content to *pdr-1* worms, these worms maintain a normal rate of oxygen consumption (Fig. 3e). This indicates that mitochondrial mass does not necessarily predict oxygen consumption. Similarly, *pink-1* worms were found to have normal levels of ATP (Fig. 3f). Since *pink-1* worms have more mitochondria than wild-type worms, this indicates that oxygen consumption and ATP production per mitochondria are both decreased in *pink-1* worms, suggesting that like *pdr-1* mutants, *pink-1* worms also accumulate dysfunctional mitochondria.

### *djr-1.1* mutants exhibit mitochondrial fragmentation

**Figure 4:**
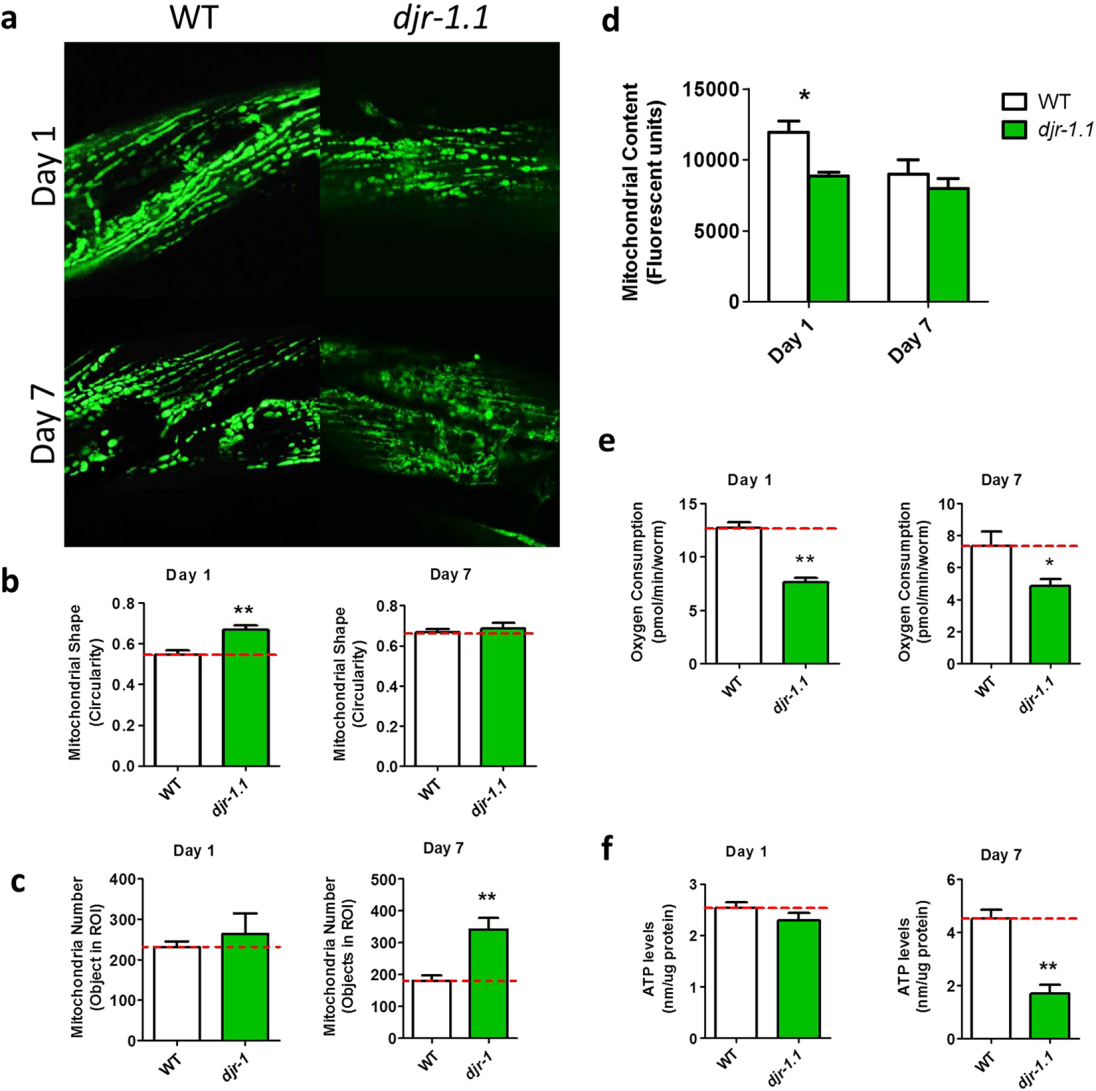
*djr-1.1* mutants exhibit mitochondrial fragmentation. (**a**) Mitochondria in *djr-1.1* mutants show increased fragmentation compared to wild-type worms. Consistent with this observation, the mitochondria in *djr-1.1* mutants are more circular than those of wild-type worms (**b**), and there is an increase in the number of mitochondria at day 7 of adulthood (**c**). In contrast, total mitochondrial content is increased on day 1 of adulthood (**d**). Oxygen consumption is decreased at both time points in *djr-1.1* mutants (**e**) leading to decreased levels of ATP (**f**). Overall, an increase in mitochondrial fragmentation in *djr-1.1* mutants results in decreased levels of ATP. Error bars indicate SEM. *p < 0.05, **p < 0.01, ***p < 0.001.

In contrast to *pdr-1* and *pink-1* mutants, *djr-1.1* worms showed marked differences in mitochondrial morphology on day 1 of adulthood. As previously observed in cell culture models^27^, we found that *djr-1.1* mutants exhibit increased mitochondrial fragmentation. While wild-type worms exhibit elongated, parallel mitochondrial networks, in *djr-1.1* mutants spherical mitochondria predominate (Fig. 4a), resulting in a significant increase in mitochondria circularity (Fig. 4b). The increase in mitochondrial fragmentation results in a higher number of mitochondria in *djr-1.1* worms compared to wild-type (Fig. 4c) but decreased total mitochondrial content as measured by integrated density of GFP (Fig. 4d). The altered mitochondrial morphology in *djr-1.1* mutants also affects mitochondrial function: *djr-1.1* mutants have a decreased rate of oxygen consumption (Fig. 4e) and decreased levels of ATP (Fig. 4f).

### *djr-1.1* mutants have increased sensitivity to oxidative stress

**Figure 5:**
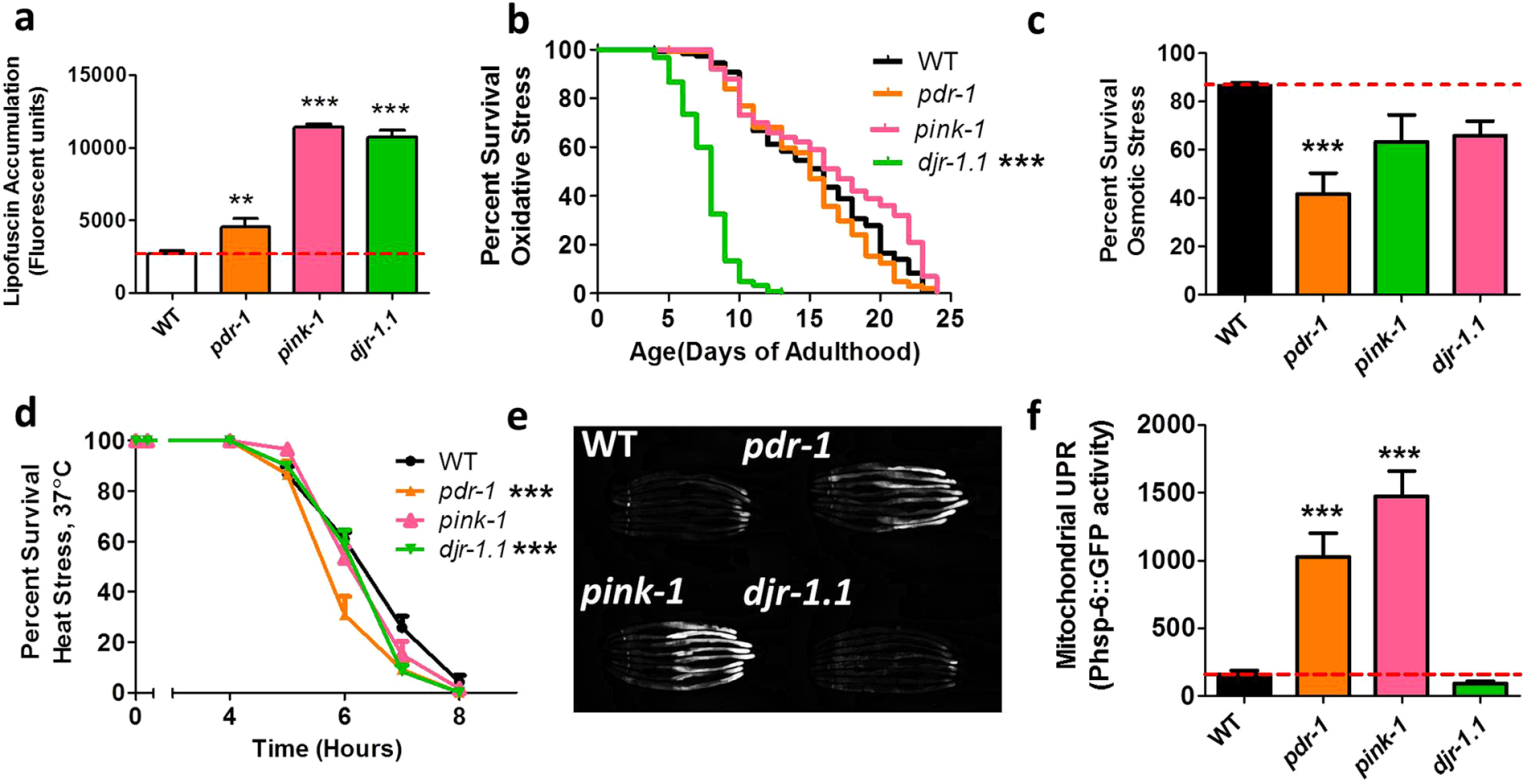
*pdr-1* and *pink-1* mutants exhibit activation of the mitochondrial unfolded protein response (mitoUPR). (**a**) *pdr-1, pink-1* and *djr-1.1* mutants all exhibit increased accumulation of lipofuscin, indicating increased levels of oxidative damage. (**b**) Measuring sensitivity to oxidative stress induced by exposing worms to 2 mM paraquat beginning at day 1 of adulthood revealed that *djr-1.1* mutants have increased sensitivity to oxidative stress. Exploring sensitivity to osmotic stress (**c**) and heat stress (**d**) showed that *pdr-1* mutants have increased sensitivity to both types of stress, while *djr-1.1* mutants have increased sensitivity to heat stress. (**e**,**f**) Both *pdr-1* and *pink-1* mutants exhibit activation of the mitoUPR as measured using a *Phsp-6::GFP* reporter strain. Error bars indicate SEM. **p < 0.01, ***p < 0.001.

Disruptions of mitochondrial function can lead to an over production of reactive oxygen species (ROS), which in turn can cause increased accumulation of oxidative damage and increased sensitivity to oxidative stress. To measure ROS levels in young worms, we stained the PD mutant worms with the ROS-sensitive dye dihydroethidium (DHE), we crossed the PD mutants to a genetically-encoded biosensor for glutathione redox potential (Grx1-roGFP2^28^), and measured protein carbonylation as a measure of oxidative damage. We did not observe any significant differences from wild-type worms in any of the PD mutants (Supplementary Fig. S4a–c). To quantify oxidative damage in aged PD mutants, we measured the levels of lipofuscin, which consists of oxidized proteins and lipids that emit a fluorescent signal, which increases with age^29^. We found that at day 10 of adulthood *pdr-1, pink-1* and *djr-1.1* mutants exhibited increased levels of lipofuscin fluorescence compared to wild-type worms, which is consistent with an increase in oxidative damage (Fig. 5a).

To test sensitivity to oxidative stress, worms were exposed to paraquat, a compound which acts to increase intracellular concentrations of superoxide primarily in the mitochondria. We found that on day 1 of adulthood, *djr-1.1* worms have increased sensitivity to chronic oxidative stress while *pdr-1* and *pink-1* worms do not differ from wild-type worms (Fig. 5b). As oxidative stress has been shown to change with age^30^, we next sought to determine whether *pdr-1* and *pink-1* mutants develop increased sensitivity to oxidative stress later in life. We examined paraquat sensitivity on day 7 of adulthood and observed a mild increase in sensitivity to oxidative stress in *pink-1* worms but not *pdr-1* worms compared to wild-type worms (Supplementary Fig. S4d).

To determine whether the PD mutants are sensitive to other forms of stress, we assayed sensitivity to both osmotic and heat stress. Both of these forms of stress induce protein misfolding, which has been associated with PD. Sensitivity to osmotic stress was assayed by exposing worms to NGM plates containing 550 mM NaCl (11X salt concentration in normal NGM plate), while heat stress was induced by incubating the animals at 37 °C. We found that *pdr-1* mutants have significantly increased sensitivity to both osmotic stress and heat stress (Fig. 5c,d), *pink-1* mutants exhibited a trend towards increased sensitivity to osmotic and heat stress that failed to reach significance (Fig. 5c,d), and *djr-1.1* exhibited a trend towards increased sensitivity to osmotic stress and significantly decreased survival under heat stress (Fig. 5c,d).

### *pdr-1* and *pink-1* mutants exhibit activation of the mitochondrial unfolded protein response

Since all three PD mutants showed increased sensitivity to stress, we next sought to determine whether these mutants exhibit activation of stress response pathways. Initially, we examined the mitochondrial unfolded protein response (mitoUPR), which is activated by disruption of protein homeostasis in the mitochondria. Since *pdr-1* mutants and *pink-1* mutants both accumulate dysfunctional mitochondria with age, we hypothesized that these worms would exhibit activation of the mitoUPR. To measure the activation of the mitoUPR, we crossed the PD mutant strains to a *Phsp-6::GFP* reporter strain (HSP-6 is a chaperone protein whose expression is upregulated by the mitoUPR)^31^. We found that the mitoUPR is activated in *pdr-1* and *pink-1* mutants (Fig. 5e,f).

### ATFS-1 is required for the normal longevity of *pdr-1* mutants

To determine if the upregulation of the mitoUPR acts to mitigate the detrimental effects of mutations in *pdr-1* and *pink-1*, we examined the effect of blocking the mitoUPR. We crossed *pdr-1* and *pink-1* mutants with an *atfs-1* deletion mutant since the transcription factor ATFS-1 is required for the mitoUPR^32^. We then compared physiologic rates between the PD mutants in a wild-type or *atfs-1* mutant background. Note that we did not examine the effect of *atfs-1* deletion in *djr-1.1* mutants because we did not observe activation of the mitoUPR in this strain. As a result, for the remaining experiments we focused on the effect of *atfs-1* deletion in *pdr-1* and *pink-1* mutants.

**Figure 6:**
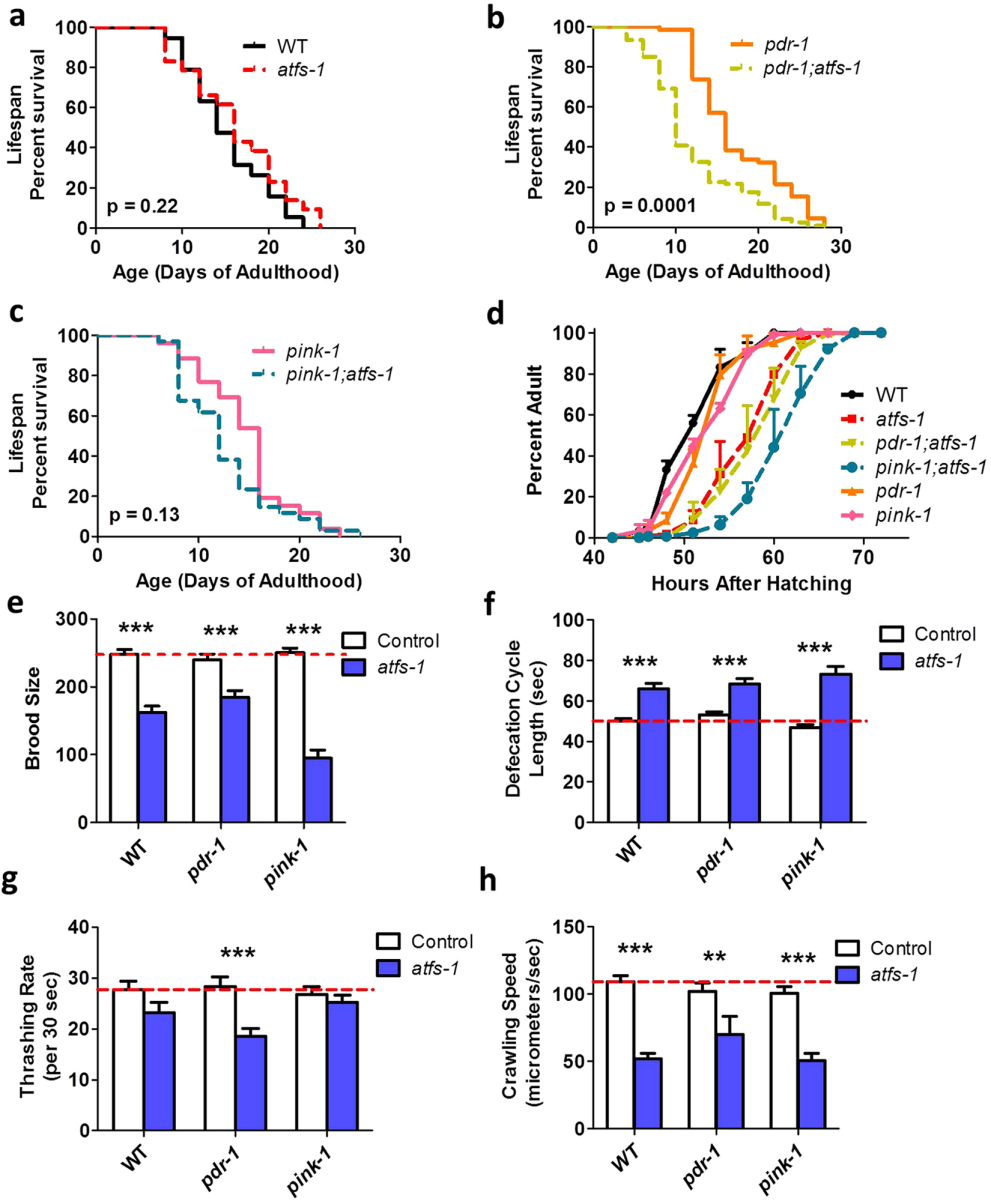
ATFS-1 is required for the normal lifespan of *pdr-1* and *pink-1* mutants. While *atfs-1* mutants live as long as wild-type worms (**a**), deletion of *atfs-1* decreases the lifespan of *pdr-1* mutants (**b**) but does not significantly decrease the lifespan of *pink-1* worms (**c**). The loss of *atfs-1* slows development time (**d**), decreases fertility (**e**), and slows the rate of defecation (**f**) in all backgrounds. The *atfs-1* deletion significantly slowed the thrashing rate of *pdr-1* mutants (**g**), and decreased crawling speed in wild-type, *pdr-1* and *pink-1* worms (**h**). Error bars indicate SEM. *p < 0.05, **p < 0.01, ***p < 0.001.

While the *atfs-1* mutation did not affect lifespan in wild-type worms, deletion of *atfs-1* significantly decreased the lifespan of *pdr-1* mutants (Fig. 6a–c). Post-embryonic development time was slowed by the *atfs-1* mutation in wild-type, *pdr-1* and *pink-1* worms (Fig. 6d), but notably there was a significant interaction between *pink-1* and *atfs-1* as *pink-1;atfs-1* worms developed slower than *atfs-1* or *pink-1* mutants alone. Fertility, as measured by brood size, was decreased by *atfs-1* deletion in wild-type, *pdr-1* and *pink-1* worms (Fig. 6e), but again to the greatest extent in *pink-1;atfs-1* double mutants. Deletion of *atfs-1* also slowed the defecation rate in all three strains (Fig. 6f). Finally, we found that the rate of movement in liquid (thrashing rate) was significantly decreased in *pdr-1;atfs-1* mutants compared to *pdr-1* mutants (Fig. 6g), while movement on solid plates was decreased in WT, *pdr-1* and *pink-1* mutants by the loss of *atfs-1* (Fig. 6h). Overall, the loss of *atfs-1* affects multiple physiologic rates, and in the case of lifespan, development time, fertility and thrashing it unmasks deficits that are present only in the PD mutant strains.

### Loss of ATFS-1 disrupts mitochondrial function and increases sensitivity to stress

**Figure 7:**
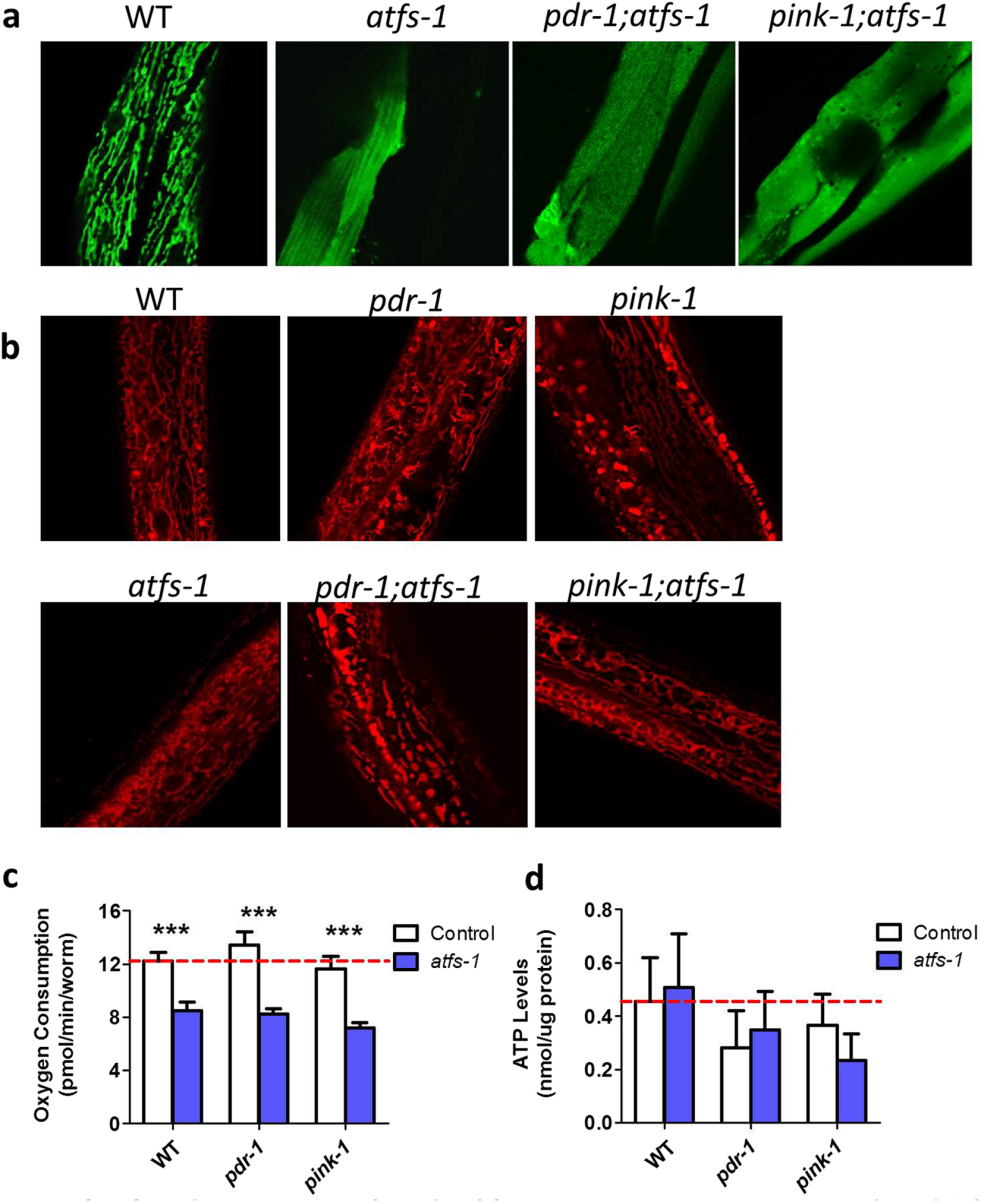
Loss of *atfs-1* causes mislocalization of mitochondrially-targeted GFP and disrupts mitochondrial function. (**a**) To examine mitochondrial morphology we crossed the *atfs-1* mutant strains to worms with mitochondrial matrix targeted GFP. We found that the loss of *atfs-1* disrupted the mitochondrial localization of the GFP resulting in diffuse fluorescence throughout the cytoplasm. (**b**) Staining mitochondria with TMRE confirms that the lack of GFP localization to mitochondria in *atfs-1* deletion mutant background does not result from the absence of mitochondria. Deletion of *atfs-1* resulted in decreased oxygen consumption in all backgrounds (**c**) but did not significantly affect the levels of ATP (**d**) Error bars indicate SEM. ***p < 0.001.

Having shown that disruption of *atfs-1* affects physiologic rates in PD mutants, we next sought to determine the effect on mitochondrial morphology and function. We crossed *pdr-1;atfs-1* and *pink-1;atfs-1* double mutants to *Pmyo-3::mito:GFP* worms to visualize mitochondrial morphology. Surprisingly, we found that in an *atfs-1* mutant background the mitochondrial matrix-targeted GFP exhibited diffuse cytoplasmic fluorescence (Fig. 7a). To determine whether this resulted from the absence of mitochondria or a mislocalization of the mitochondria-targeted GFP, we stained worms with the mitochondria specific dye tetramethylrhodamine ethyl ester (TMRE). TMRE successfully delineated the mitochondria in the *atfs-1* mutant background (Fig. 7b). This suggests that the *atfs-1* mutant causes mislocalization of the mitochondria-matrix targeted GFP. Examination of mitochondrial function revealed that the *atfs-1* mutation caused a decrease in oxygen consumption in wild-type, *pdr-1* and *pink-1* worms (Fig. 7c), however it did not significantly alter ATP levels (Fig. 7d).

**Figure 8:**
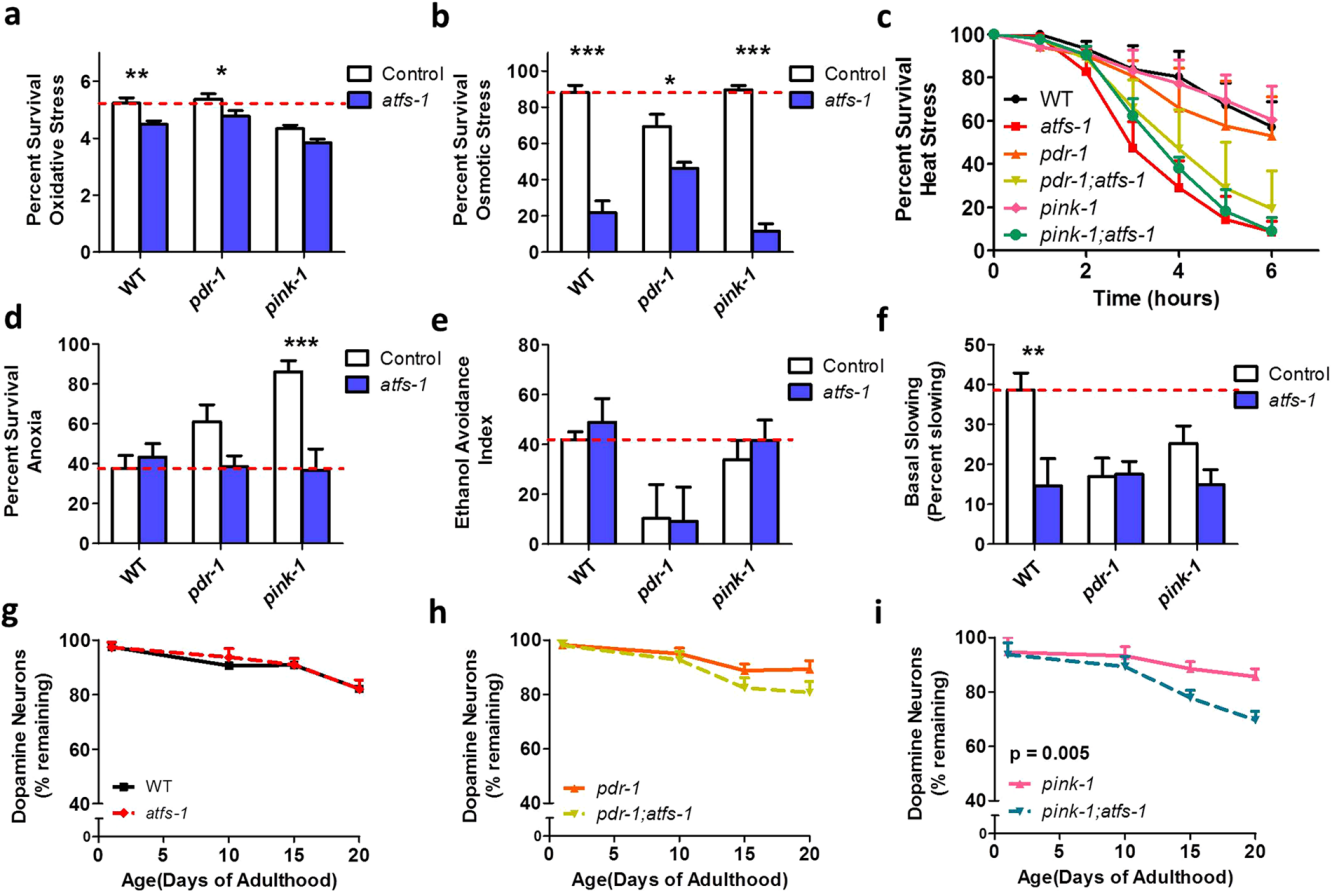
The mitoUPR is required for the survival of dopamine neurons in *pdr-1* and *pink-1* mutants. Deletion of *atfs-1* results in increased sensitivity to oxidative stress (3 mM paraquat, **a**) osmotic stress (550 mM NaCl, **b**) heat stress (37 °C, **c**) and anoxia (**d**) The loss of *atfs-1* does not exacerbate deficits in ethanol avoidance (**e**) or basal slowing (**f**) in the Parkinson’s disease mutants, but did induce a basal slowing deficit in wild-type worms. (**g**) Deletion of *atfs-1* does not decrease the survival of dopamine neurons in wild-type worms. (**h**) *pdr-1;atfs-1* double mutants exhibit a trend towards decreased numbers of dopamine neurons compared to *pdr-1* mutants. (**i)** Loss of *atfs-1* accelerated dopamine neuronal loss in *pink-1* mutants. Error bars indicate SEM. *p < 0.05, **p < 0.01, ***p < 0.001.

Next, we examined the effect of the *atfs-1* mutation on resistance to stress in the PD mutant strains. We found that deletion of *atfs-1* increased sensitivity to 3 mM paraquat oxidative stress (Fig. 8a), 550 mM NaCl osmotic stress (Fig. 8b), 37 °C heat stress (Fig. 8c), and anoxia (Fig. 8d). Interestingly, although loss of *atfs-1* affected oxidative stress, osmotic stress, and heat stress similarly in the PD mutants and wild-type worms, it only increased sensitivity to anoxia in the PD mutants.

### Activation of the mitoUPR protects dopamine neurons from death

Finally, we determined the effect of blocking the mitoUPR on PD-like deficits in *pdr-1* and *pink-1* mutants. There was no effect of the *atfs-1* deletion on ethanol avoidance (Fig. 8e); however, it should be noted that we observed increased clumping behavior in worms with the *atfs-1* deletion, which could have impacted the outcome of this assay. In the basal slowing assay, the *atfs-1* mutation caused a significant deficit in basal slowing in a wild-type background, but did not further exacerbate basal slowing deficits present in *pdr-1* and *pink-1* worms (Fig. 8f). Quantification of dopamine neuron loss showed that loss of *atfs-1* did not affect neuronal survival in a wild-type background (Fig. 8g). *pdr-1;atfs-1* mutants show a trend towards increased dopamine neuron loss compared to *pdr-1* single mutants (Fig. 8h). *pink-1;atfs-1* worms had a significantly increased rate of dopamine neuron loss compared to wild-type, *atfs-1* or *pink-1* worms (Fig. 8i). This indicates that the ability to activate the mitoUPR is required for dopamine neuron survival in *pink-1* PD mutants and that the mitoUPR can be neuroprotective in genetic models of PD.

## Discussion

In order to identify and test disease modifiers for Parkinson’s disease, it is essential to have accurate animal models with robust phenotypic deficits that are analogous to symptoms present in the human disease. If this can be accomplished in a simple invertebrate organism, it becomes possible to screen for modifiers rapidly and cost-effectively. As yeast do not have homologs to *PRKN* or *PINK1* ^33^, *C. elegans* represents the simplest genetic model organism with homologs to *PRKN, PINK1* and *DJ-1*. Accordingly, we characterized phenotypic deficits in *C. elegans* mutants with deletions in each of these mitochondria-associated genes that have been implicated in PD.

*pdr-1* mutants have decreased lifespan and increased sensitivity to rotenone^34^, decreased survival against bacterial pathogens^35^, increased sensitivity to heat stress and paraquat-induced oxidative stress^36^, and increased accumulation of manganese, which has been associated with the development of PD^37^. In addition, *pdr-1* RNAi decreases the survival of dopamine neurons in worms expressing α-synuclein in their dopamine neurons^38^ or in worms expressing LRRK2 treated with rotenone^39^. Here, we found that the *pdr-1* mutation alone is not sufficient to decrease the survival of dopamine neurons. Nonetheless, *pdr-1* mutants exhibit deficits in two dopamine-dependent behaviors: ethanol avoidance and basal slowing. This suggests that loss of *pdr-1* causes dysfunction of the dopamine neurons but not death.

*pink-1* mutants exhibit increased sensitivity to paraquat-induced oxidative stress^36,40^, heat stress^36^ and bacterial pathogenicity^35^. As with *pdr-1*, knocking down *pink-1* using RNAi decreased the survival of dopamine neurons exposed to either α-synuclein or LRRK2 plus rotenone^38,39^. In this work, we find that *pink-1* worms show a significant reduction in basal slowing, but exhibit a wild-type survival of dopamine neurons. Again, this suggests that the deficit in dopamine-dependent behavior in *pink-1* worms results from neuronal dysfunction.

Consistent with the role of DJ-1 in sensing and protecting against oxidative stress, *djr-1.1* worms have decreased survival compared to wild-type worms when exposed to glyoxals, while *djr-1.2* worms exhibit a more rapid loss of dopamine neurons under the same conditions^41^. DJR-1.2 was also shown to protect dopamine neurons from manganese toxicity^22^. We found that *djr-1.1* mutants exhibit increased sensitivity to paraquat induced oxidative stress, but do not show any significant deficits in dopamine-dependent behavior. In addition, under unstressed conditions, neither *djr-1.1* nor *djr-1.2* mutants exhibit decreased survival of dopamine neurons.

Combined our results indicate that disruption of mitochondria-associated genes implicated in PD, cause multiple mild phenotypic deficits but no overt neuronal loss. Interestingly, although Parkin, PINK1 and DJ-1 have been shown to act together for specific functions^42,43^, the fact that we observe different phenotypes in each of the deletion mutants highlights the fact that these proteins also have functions that are independent of the other two proteins.

### Parkinson’s disease mutants exhibit age-dependent alterations in mitochondrial morphology and function

In characterizing the three PD mutants, we observed differences in both the form and function of the mitochondria. In *djr-1.1* mutants, mitochondrial fragmentation was increased by day 1 of adulthood, leading to the observed decrease in oxygen consumption and lower levels of ATP. Cell culture models of DJ-1 deficiency have also been shown to have an increase in mitochondrial fragmentation^27^. In *pdr-1* and *pink-1* mutants, we found that total mitochondrial content was similar to wild-type worms on day 1 of adulthood, but both mutants showed an accumulation of mitochondria with increasing age. *pdr-1* and *pink-1* mutants were previously shown to exhibit increased mitochondrial content in the intestine^36^. In addition to increased mitochondrial abundance, *pdr-1* and *pink-1* worms also showed altered morphology, as has been previously noted^36,44^. Two previous studies also examined oxygen consumption with differing results: one found that oxygen consumption was wild-type in both mutants^44^, while the other observed increased oxygen consumption^36^. In agreement with the former study, we observed normal oxygen consumption on day 1 of adulthood. However, similar to the latter study, we found that oxygen consumption is increased in *pdr-1* mutants by day 7 of adulthood. Surprisingly, *pdr-1* worms showed decreased levels of ATP despite the increased rate of oxidative phosphorylation, suggesting that these mitochondria are dysfunctional. In *pink-1* mutants oxygen consumption and ATP levels were still equivalent to wild-type at day 7 of adulthood, despite the increase in mitochondrial content. This indicates that the ATP produced per mitochondria is decreased in *pink-1* mutants, which is consistent with previous observations of proton leak in *pink-1* mutants^44^. A reduction in mitochondrial cristae length in *pink-1* mutants^40^ may also contribute to the decreased ability to produce ATP.

While it would have been more informative to examine maximal respiration and ATP-linked respiration in these mutants, these measurements are challenging in whole worms. Nonetheless, it was previously shown that both maximal and ATP-linked oxygen consumption rates are equivalent to wild-type in *pdr-1* and *pink-1* mutants^44^. An alternative approach would be to measure these outputs in dissociated primary cells; however, it is unclear to what extent these readings would reflect what is happening in an intact, whole organism.

### Activation of the mitochondrial unfolded protein response promotes dopamine neuronal survival

The accumulation of dysfunctional mitochondria with age in *pdr-1* and *pink-1* mutants resulted in the activation of the mitoUPR. To determine if mitoUPR activation was masking deficits caused by the *pdr-1* and *pink-1* mutation, we disrupted the mitoUPR using an *atfs-1* deletion mutation. Under normal conditions ATFS-1 is imported into the mitochondria and subsequently degraded. However, under conditions of mitochondrial stress, ATFS-1 import efficiency is reduced allowing this protein to build up in the cytosol, where it can be transported into the nucleus and activate the mitoUPR^32^. We found that disrupting *atfs-1* significantly decreased the survival of *pdr-1* mutants and accelerated the loss of dopamine neurons in *pink-1* worms. In contrast, the loss of *atfs-1* did not affect the lifespan or dopamine neuronal survival in wild-type worms. The loss of *atfs-1* did not affect ATP levels in any of the mutants, indicating that the effect of this mutation on other phenotypes is not mediated through a decrease in ATP levels. Overall, our results indicate that the activation of the mitoUPR in *pdr-1* and *pink-1* worms is protecting these mutants from the detrimental effects of the abnormal form and function of the mitochondria.

The presence of mtDNA deletions can activate the mitoUPR^45^, and both activation of the mitoUPR and deletion of *pdr-1* allow for the accumulation of mtDNA bearing a deletion^46^. In addition, PDR-1 can modulate the degree of heteroplasmy for an mtDNA truncation^47^, and reducing the levels of PINK-1 decreases the removal of mtDNA damage^48^. While on the surface, it would seem to be detrimental to increase the amount of damaged mtDNA through the activation of the mitoUPR, the fact that blocking the mitoUPR in *pdr-1* and *pink-1* mutants has detrimental effects suggests that this is beneficial for organismal and dopamine neuron survival. Further studies will be required to elucidate the precise mechanisms underlying the observed neuroprotection.

## Conclusions

Overall our results show that there are multiple mild phenotypic deficits present in *pdr-1, pink-1*, and *djr-1.1* mutants. All three mutants exhibit abnormalities in both mitochondrial form and function: *djr-1.1* mutants primarily show mitochondrial fragmentation while *pdr-1* and *pink-1* mutations exhibit an accumulation of dysfunctional mitochondria. Both *pdr-1* and *pink-1* mutants show an activation of the mitoUPR, which is neuroprotective and mitigates the detrimental effect of the *pdr-1* and *pink-1* mutations.

## Methods

### Strains

N2(WT)

JVR209 *cat-2(e1112)*

BY250 *vtIs7[Pdat-1::GFP(pRB490)]*

JVR122 *bcIs78[Pmyo-3::mitoGFP]*

JVR121 *zcIs13[Phsp-6::GFP]*

JVR392 *dvIs19[Pgst-4::GFP]*

JVR043 *pdr-1(gk448)*

JVR036 *pdr-1(gk448); vtIs7[Pdat-1:GFP(pRB490)]*

JVR310 *pdr-1(gk448); bcIs78[Pmyo-3::mitoGFP]*

JVR345 *pdr-1(gk448); zcIs13[Phsp-6::GFP]*

JVR392 *pdr-1(gk448); dvIs19[Pgst-4::GFP]*

MQ1775 *pink-1(ok3538)*

MQ1794 *pink-1(ok3538); vtIs7[Pdat-1:GFP(pRB490)]*

JVR309 *pink-1(ok3538); bcIs78[Pmyo-3::mitoGFP]*

JVR346 *pink-1(ok3538); zcIs13[Phsp-6::GFP]*

JVR391 *pink-1(ok3538); dvIs19[Pgst-4::GFP]*

JVR066 *djr-1.1(tm918)*

JVR220 *djr-1.1(tm918); vtIs7[Pdat-1::GFP(pRB490)]*

JVR311 *djr-1.1(tm918); bcIs78[Pmyo-3::mitoGFP]*

JVR347 *djr-1.1(tm918); zcIs13[Phsp-6::GFP]*

JVR394 *djr-1.1(tm918); dvIs19[Pgst-4::GFP]*

JVR496 *djr-1.2 (tm1346)*

JVR374 *djr-1.2(tm1346); vtIs7[Pdat-1::GFP(pRB490)]*

JVR455 *atfs-1(gk3094)*

JVR481 *atfs-1 (gk3094); vtIs7[Pdat-1::GFP(pRB490)]*

JVR482 *pdr-1(gk448); atfs-1 (gk3094); vtIs7[Pdat-1::GFP(pRB490)]*

JVR483 *pink-1(ok3538); atfs-1 (gk3094); vtIs7[Pdat-1::GFP(pRB490)]*

JVR484 *atfs-1 (gk3094); bcIs78[(Pmyo-3::mitoGFP)]*

JVR485 *pdr-1(gk448); atfs-1 (gk3094); bcIs78[(Pmyo-3::mitoGFP)]*

JVR486 *pink-1(ok3538); atfs-1 (gk3094); bcIs78[(Pmyo-3::mitoGFP)]*

JVR135 *jrIs2[Prpl-17::Grx1-roGFP2]*

JVR498 *pdr-1(gk448); jrIs2[Prpl-17::Grx1-roGFP2]*

JVR499 *pink-1(ok3538); jrIs2[Prpl-17::Grx1-roGFP2]*

JVR500 *djr-1.1(tm918); jrIs2[Prpl-17::Grx1-roGFP2]*

All strains were maintained at 20 °C on NGM plates seeded with OP50 bacteria. All strains were outcrossed to N2 (wild-type) three to six times to obtain a uniform strain background.

#### Generation of strains with multiple mutations

Double/triple mutants were generated as previously described^49^. Homozygosity of fluorescent transgenes was determined by counting 20–30 worms in three consecutive generations to ensure 100% fluorescence.

### Physiologic rates

#### Lifespan

Lifespan was measured on plates containing 25 μM 5-fluoro-2′-deoxyuridine (FUdR) to limit the growth of progeny and minimize the impact of the FUdR on lifespan^50^. Animals were transferred to fresh plates after 4 days because this concentration of FUdR does not completely prevent the development of progeny to adulthood in the first generation. From this point on, worms were transferred to fresh plates weekly. Viability was assessed every 2 days by gentle prodding. Worms that either had internal hatching of progeny or expulsion of internal organs were not counted as deaths.

#### Defecation rate

Defecation cycle length was determined by measuring the time between consecutive pBoc contractions in day-1 adult worms with at least 10 worms per replicate. To minimize the effects of ambient laboratory temperature, defecation was measured on water filled chambers that had been incubated at 20 °C and the lids of the plates containing the worms were not removed.

#### Postembryonic development time

Post-embryonic development (PED) time was measured by transferring eggs to an NGM plate. After 3 h, newly hatched L1 worms (20–30 worms per replicate) were transferred to a new NGM plate. The time from hatching to the young adult transition was measured as the PED time.

#### Fertility

Brood size was measured by placing individual worms at the L4 stage onto NGM plates followed by daily transfers to new plates for three days. The resulting progeny was allowed to develop to adulthood before counting (5 worms per replicate).

#### Rate of movement

Thrashing rate was assessed by measuring swimming behavior in liquid using video-tracking and computer analysis. Approximately 50 worms were placed in M9 buffer (22 mM KH_2_PO_4_, 34 mM K_2_HPO_4_, 86 mM NaCl, 1 mM MgSO_4_) on a clean NGM plate. Videos were taken with an Allied Vision Tech Stingray F-145 B Firewire Camera (Allied Vision, Exton, PA, USA) at 1024 × 768 resolution, 8-bit using the MATLAB image acquisition toolbox. Analysis was performed using wrMTrck plugin for ImageJ (publically available at http://www.phage.dk/plugins).

#### Stress resistance assays: Heat stress, Oxidative stress, Osmotic stress, Anoxia

Stress assays were performed in pre-fertile young adult worms as we have previously described^51^. Sensitivity to heat stress was determined through hourly assessment of survival at 37 °C. Sensitivity to chronic oxidative stress was determined through exposure to plates containing 2–3 mM paraquat (methyl viologen, Sigma) beginning at day-1 of adulthood. Plates also contained 100 µM FUdR to prevent paraquat-induced internal hatching of progeny. Survival was monitored daily until death. Sensitivity to osmotic stress was determined by transferring day 1 adult worms to NGM plates containing 500 mM NaCl. The survival of worms was determined after 24 h. All stress assays were completed on day-1 of adulthood and included a minimum of 30 worms per replicate.

We assessed nematode resistance to low oxygen environments by utilizing the Becton-Dickinson Bio-Bag Environmental Chamber. Anaerobic atmosphere is achieved within the sealed Bio-Bag using a self-contained generator and resazurin indicator made up of an ampule of HCL solution and two gas-generating tablets. The ampule was crushed, and the acid reacted with the tablets resulting in a gas mixture that absorbs oxygen in the presence of palladium. 30–50 animals were moved to freshly seeded NGM plates and placed in the anoxic chamber for 50 hours. Recovery was allowed for 24 hours, at which time the survival percentage was assessed.

#### Measurement of fluorescent reporter activity

Reporter activity in adult worms was assessed through measurement of whole worm florescence as previously described^52^. For each biological replicate, 10 animals were paralyzed with 2 mM levamisole, mounted on an NGM plate and fluorescent images were captured using an AVT Stingray F145B camera and VimbaViewer 1.1.2 software. ImageJ was used to measure the average pixel intensity.

### Dopamine-dependent behaviors

#### Basal slowing

Approximately 50 worms at day 3 of adulthood were washed in M9 buffer to remove any residual bacteria. These cleaned animals were then transferred to either unseeded NGM plates or NGM plates seeded with OP50 bacteria covering the entire plate. After 5 min, videos of the entire plate were recorded for 1 min with an Allied Vision Tech Stingray F-504 B Firewire Camera and a Navitar Zoom 7000 lens (Navitar, Tokyo, Japan) using and the MATLAB image acquisition tool. Recordings were processed using the wrMTrck plugin for ImageJ. Basal slowing was calculated as the difference in rate of movement on food versus off food divided by the rate of movement off food. Crawling speed was calculated from data collected from the unseeded “off-food” NGM plate.

#### Ethanol avoidance assay

Day-1 adult worms were transferred to assay plates, which were divided into four sectional quadrants: two seeded with 150 μl ethanol (applied in 50 μl increments over 1 hour) and the others without. Worms were cleaned in M9 and then plated in minimal liquid in the center of the assay plate and allowed to move for 30 min at which point the entire plate is imaged and the worms are scored for their quadrant of preference. Ethanol avoidance is calculated as:

((number of worms in control quadrants)−(number of worms in ethanol quadrants))/total number of worms.

#### Measurement of reactive oxygen species

The levels of ROS were determined using the ROS-sensitive dye dihydroethidium (DHE) as described previously^53^. 50 worms were incubated in 750 µL of 3 µM DHE dissolved in DMSO for 30 minutes while shaking. After washing in M9 buffer (3 time, 10 minutes), worms were allowed to crawl across an empty NGM plate for 15 minutes prior to imaging. As an alternative approach to measuring ROS levels we used a strain expressing the ratiometric biosensor Grx1-roGFP2^28^. We used a Nikon A1r confocal laser scanning microscope mounted upon a Nikon A1R Ti inverted epifluorescence microscope set up and induced excitation using 405 nm and 488 nm solid state lasers. Age synchronized worms expressing Grx1-roGFP2 were paralyzed with 10 mM levamisole and mounted onto a slide with a 1% agar pad. Animals were imaged using a 40 × 0.95 N.A. objective coupled to a single emission filter (525/50 nm) to create a composite 488 nm/405 nm ratio image. Composite ratio images were then analyzed using ImageJ to subtract background and quantify particle number and intensity.

#### Measurement of oxidative damage

To quantify oxidative damage in young worms, we measured protein carbonylation using an OxyBlot protein oxidation detection kit (Millipore) according to the manufacturer’s instructions, as we have done previously^54^. To quantify oxidative damage in old worms, we measured lipofuscin levels. Synchronized day 1 adult animals were plated on 25 μM FUdR plates and aged to day 10 of adulthood. Worms were then transferred to a clean 60 mm NGM plate and paralyzed using 2 mM levamisole. Images of 10 whole worms were captured using a Nikon SMZ 1500 dissecting microscope (Tokyo, Japan) with an Allied Vision Tech Stingray F-145 B Firewire Camera using Vimba image acquisition software (Allied Vision, Exton, PA, USA) and the fluorescence was quantified using ImageJ.

#### Degeneration of dopamine neurons

The degeneration of dopamine neurons was monitored by expressing GFP specifically in dopamine neurons under the *dat-1* dopamine transporter promoter. It was previously shown that treatments that cause a loss of GFP-positive neurons cause a corresponding loss of dopamine neuron cell bodies. Synchronized *Pdat-1:GFP* worms were plated onto 25 μM FUdR agar plates to be observed at increasing ages. At each time point 10–15 worms were mounted onto a 1% agar pad on a glass slide, immobilized using 2 mM levamisole, and enclosed with a coverslip. Imaging of immobilized animals was carried out with an Axioplan 2 inverted fluorescence microscope (Zeiss, Oberkochen, Germany). Quantification of dopamine neurons was done in a blinded manner.

#### Oxygen consumption measurements

Basal oxygen consumption rate was measured using a Seahorse XF_e_96 analyzer (Seahorse bioscience Inc., North Billerica, MA, USA). Synchronized worms were aged to the time point of interest and then cleaned in M9. Cleaned nematodes were pipetted in calibrant (at 30–90 worms per well) into a Seahorse 96-well plate. Oxygen consumption was measured six times and rates of respiration were normalized to the number of worms in each individual well. The plate readings were begun within 20 minutes of introduction of the worms into the well. Reading from each well were normalized relative to the number of animals per well. Well probes were hydrated in 175 µL Seahorse calibrant and the machine was allowed to equilibrate to room temperature overnight proceeding this assay to prevent nematode death.

#### ATP Measurement

Approximately 200 worms were age-synchronized by transferring daily. Worms were collected in de-ionized water, washed, and were freeze-thawed 3 times. The resulting pellet was sonicated in a Bioruptor (Diagenode) with 30 cycles of 30 seconds on, 30 seconds off. The pellet was boiled for 15 minutes to release ATP, then spun at 4 degrees at 11,000 g for 10 minutes. The supernatant was collected and measured using a Molecular Probes ATP determination Kit (Life Technologies). Luminescence was normalized to protein content, which was measured with a Pierce BCA protein determination kit (Thermo Scientific). Statistical analysis was performed using Graphpad software.

#### Mitochondrial Imaging and quantification of mitochondrial morphology

Worms were crossed to *Pmyo-3::mito-GFP* worms and age-synchronized. For imaging, worms were transferred to an agar pad mounted on a slide. Worms were subsequently paralyzed with 10 µM levamisole and cover-slipped. Images were captured by confocal microscopy (Nikon A1R Ti) at 60x. A z-stack of 17 images spaced 0.125 µm apart was collected. For the representative images shown in the figures, a maximum image projection was created in Nikon Elements Basic Research (v.4.6) to compress z-stacks into a single image. All imaging conditions were kept the same for all images. For quantification, a single representative slice for each z-stack was used to avoid the complication of mitochondria being present in two planes. This slice was made binary using the Nikon Elements thresholding tool. First, a background subtraction of a constant 50 was applied. A threshold for the GFP signal was determined by applying the average threshold for mask creation of control images and was applied to all images. Mitochondrial circularity, number, and area were measured using the measure objects tool in Nikon Elements after the threshold was applied. For mitochondrial circularity, raw numbers were exported to Microsoft Excel and averages were calculated prior to statistical analysis in Graphpad. All other calculations were exported directly to Graphpad for analysis.

#### Statistical analysis

All data was found to be parametic, following a normal distribution. Homoschedasticity was confirmed using Bartlett’s test. Survival plots were compared using the log-rank test. For analyses involving multiple groups, either a one way ANOVA or two way ANOVA was used to assess significance followed by a Bonferroni post-hoc test for detecting specific differences between groups. For lifespan assays, we completed three replicates with 40 worms per replicate. For brood size assays, we completed three replicates with 5 worms per replicate. For post-embryonic development time assays, we completed three replicates with 25 worms per replicate. For defecation assays, we completed three replicates with 5–10 worms per replicate. For thrashing assays, we completed three replicates with 50 worms per replicate. For crawling speed assays, we completed three replicates with 10 worms per replicate. For ethanol avoidance assays, we completed eight replicates with 50 worms per replicate. For basal slowing assays, we completed three replicates with 10 worms per replicate. For dopamine neurodegeneration assays, we performed three replicates with a minimum of 10 different animals per time point. For mitochondrial morphology assays, we completed three replicates with 3–5 worms per replicate. For oxygen consumption assays, we completed at least 10 replicates using a population of worms for each replicate. For ATP assays, we completed at least three replicates using a population of worms per replicate. For lipofuscin measurement and measurement of Phsp-6::GFP reporter activity, we performed measurements in 10 different animals. For paraquat assays, we completed three replicates with 40 worms per replicate. For osmotic stress and heat stress assays, we completed six replicates with 25 worms per replicate.

## Electronic supplementary material

Supplementary Figures
