# Supplementary material for: Activation of the mitochondrial unfolded protein response promotes longevity and dopamine neuron survival in Parkinson’s disease models
Source: Sci Rep. 2017 Nov 27;7:16441. doi: 10.1038/s41598-017-16637-2 (PMC5703891; doi:10.1038/s41598-017-16637-2)
Supplement: Supplementary file 1 — Supplementary Figures [file 41598_2017_16637_MOESM1_ESM.pdf]

Supplementary Figures for:

Activation of the mitochondrial unfolded protein response promotes longevity and dopamine neuron survival in Parkinson’s disease models

Jason F. Cooper[1], Emily Machiela[1], Dylan J. Dues[1], Katie K. Spielbauer[1], Megan M. Senchuk[1], Jeremy M. Van Raamsdonk[1,2,3]*

[1]Laboratory of Aging and Neurodegenerative Disease, Center for Neurodegenerative Science, Van Andel Research Institute, Grand Rapids MI, USA

[2]Department of Neurology and Neurosurgery, McGill University, Montreal, Quebec, Canada

[3]Metabolic Disorders and Complications Program, and Brain Repair and Integrative Neuroscience Program, Research Institute of the McGill University Health Centre, Montreal, Quebec, Canada

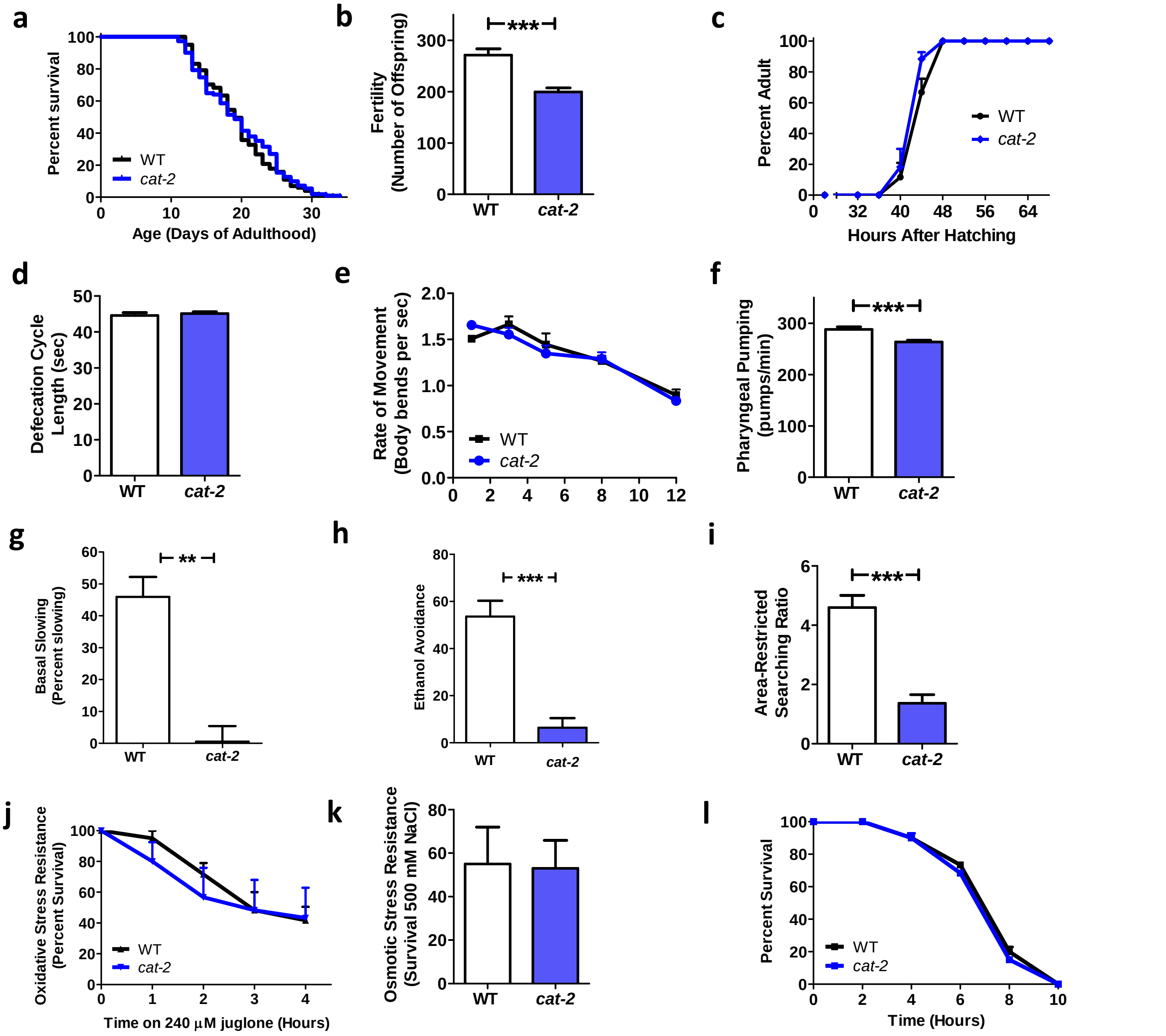

Supplementary Figure S1. Dopamine-dependent phenotypes present in *cat-2* tyrosine hydroxylase mutants. In order to determine what phenotypes result from the loss of dopamine, we compared physiologic rates, dopamine dependent behaviors and stress resistance between wild-type and *cat-2* dopamine-depletion mutants. We assessed **a)** lifespan, **b)** fertility, **c)** post-embryonic development, **d)** defecation cycle length, **e)** rate of movement, **f)** pharyngeal pumping rate, **g)** basal slowing, **h)** ethanol avoidance, **i)** area-restricted searching, **j)** oxidative stress resistance **k)** osmotic stress resistance and **l)** heat stress resistanace,. We observed marked deficits in basal slowing, ethanol avoidance and area-restricted searching with mild differences in fertility and pharygeal pumping. White indicates wild-type worms, blue indicates *cat-2* mutant worms. Error bars indicate SEM. **p<0.01, ***p<0.001.

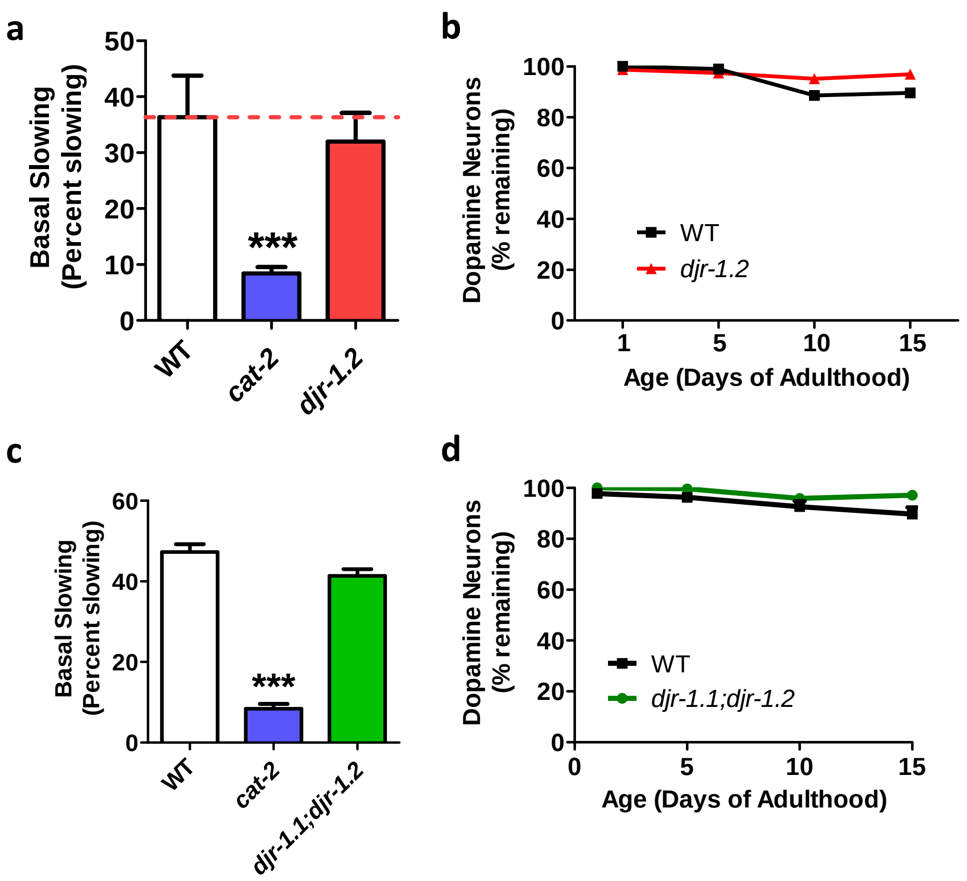

Supplementary Figure S2. *djr-1.2* and *djr-1.1;djr-1.2* mutants do not exhibit deficits in dopamine-dependent behavior or loss of dopamine neurons. a. Basal-slowing was equivalent to wild-type worms in *djr-1.2* mutants. **b.** There was no evidence of dopamine neuron loss in *djr-1.2* worms. Similarly, basal slowing (**c**) and dopamine neuron survival (**d**) were wild-type in *djr-1.1;djr-1.2* mutants. Error bars indicate SEM. *** p<0.001.

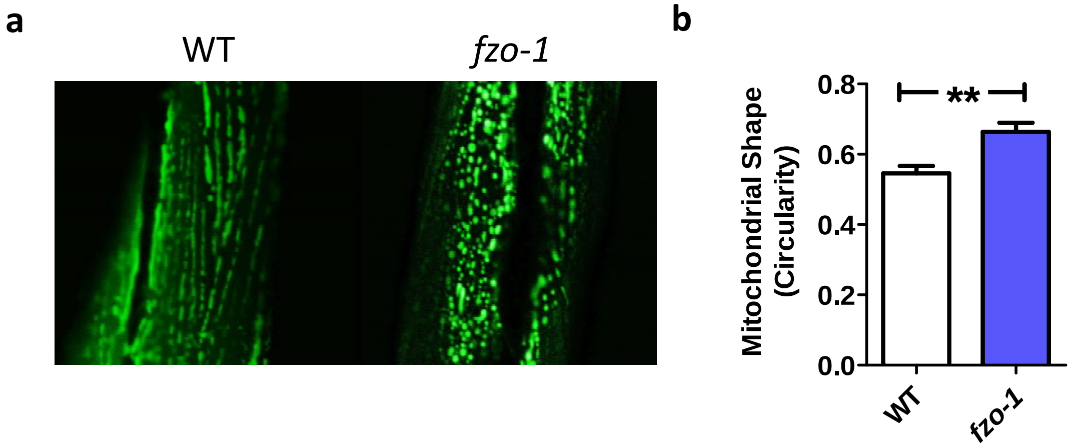

Supplementary Figure S3. Deletion of mitochondrial fusion gene *fzo-1* results in increased mitochondrial fragmentation. a. Mitochondria in *fzo-1* mutants show increased fragmentation **b.** The circularity of *fzo-1* mitochondria is significantly increased compared to wild-type. Error bars indicate SEM. **p<0.01.

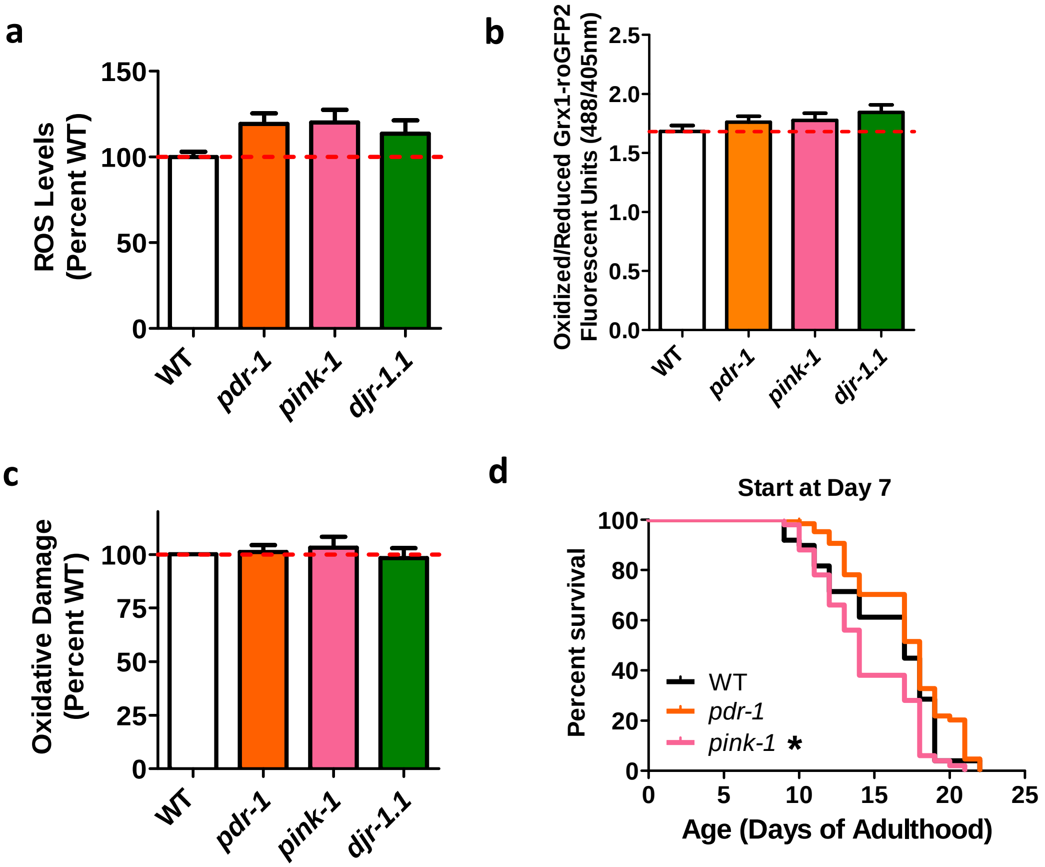

Supplementary Figure S4. Reactive oxygen species and oxidative damage are wild-type in day 1 Parkinson's disease mutants. a. The levels of ROS, as measured by dihydroethidium (DHE) fluorescence is equivalent to wild-type worms in day 1 adult *pdr-1, pink-1* and *djr-1.1* mutants. **b.** Similarly, the glutathione redox potential, as measured using a roGFP2 genetically-encoded biosensor, is also equivalent to wild-type worms in *pdr-1, pink-1* and *djr-1.1* mutants. **c.** Oxidative damage, as measured by Western blotting for carbonylated proteins, is also wild-type in day 1 adult Parkinson's disease mutants. **c.** Testing sensitivity to oxidative stress on plates containing 2 mM paraquat beginning on day 7 of adulthood reveals mildly increased sensitivity in *pink-1* mutants. Error bars indicate SEM. * $p<0.05$.
